# Supplementary material for: Therapeutic Targeting of Inflammation and Virus Simultaneously Ameliorates Influenza Pneumonia and Protects from Morbidity and Mortality
Source: Viruses. 2023 Jan 23;15(2):318. doi: 10.3390/v15020318 (PMC9966636; doi:10.3390/v15020318)
Supplement: Supplementary file 1 [file viruses-15-00318-s001.zip › viruses-2162051-supplementary.pdf]

Supporting Information for

Therapeutic targeting of inflammation and virus simultaneously ameliorates influenza pneumonia and protects from morbidity and mortality

Materials and Methods

Plaque assay for virus quantification

Plaque assay for IAV quantification was previously described elsewhere [32]. Briefly, Madin-Darby Canine Kidney (MDCK) cells were seeded in a 6-well tissue culture plate at 1.2×10^6 cells/well and incubated overnight in the cell growth media. On the day of infection, 2× Leibovitz L15 medium (ThermoFisher scientific, cat. no. 41300039) and 1.8 % low melting point agarose (Lonza, cat. no. 51101) were pre-warmed in a water bath at 37°C and 46°C, respectively until required. Each lung sample was then weighed and homogenized in serum free DMEM media using TissueLyser II (Qiagen, cat no. 85300). The homogenized tissue samples were then sonicated at 100W power in three 15-second bursts in a cup sonicator (Branson Sonic Power Company, Danbury, CT, USA) to further break up clumps of tissue and release virus. MDCK cell monolayers were washed with serum free DMEM medium leaving around 0.2 mL of media in each well. Six 10-fold serial dilutions of the homogenized lung samples were prepared in serum free DMEM supplemented with 1× PSN antibiotics and 1.5 µg/mL L-1-Tosylamide-2-phenylethyl chloromethyl ketone (TPCK)-trypsin (Sigma-Aldrich, cat. no. T1426). This will be referred to as virus growth medium. From each dilution, a 100 µl volume was added to the cell monolayer, which was then incubated for 1 h at 37°C with intermittent shaking every 15 mins. Pre-warmed 2× Leibovitz L15 medium (Invitrogen, cat. no. 41300039) and 1.8% low melting point agarose (Lonza, cat. no. 50101) were mixed in equal (1:1) ratio to prepare the agar overlay which was supplemented with TPCK- trypsin at 1.5 µg/mL. The infected monolayer was then covered with 3 mL agar overlay. After agar solidification, the tissue culture plates were incubated at 37°C, 5 % CO2 for 4 days. The cells were next fixed with 10% formalin for 30 min at room temperature and the agar plug was removed using a sterile spatula. Finally, plaques were visualized by staining with 200 µl 0.1% crystal violet followed by rinsing with water. To determine the viral titer of the sample, plaques were counted and the following equation was used.

$$\text{PFU/g lung} = \frac{\text{No. of plaques} \times \text{dilution factor} \times \text{vol. of homogenization solvent } (\mu\text{L})}{\text{vol. of viral suspension applied } (\mu\text{L}) \times \text{weight of lung tissue (g)}}$$

$TCID_{50}$ Assay for Virus Quantification

$TCID_{50}$ assay for IAV quantification has been described elsewhere [32]. Briefly, MDCK cells were seeded in a 96-well tissue culture plates at 2.5×10^4 cells per well and grown overnight in the cell growth media. Lungs were homogenized as described for the plaque assay. Eight 10-fold serial dilutions of lung homogenates were prepared in the virus growth medium and 25 µL of each dilution was inoculated onto cell monolayers in 10 replicates. After 1 h of virus adsorption, the inoculum was removed and the infected monolayer was incubated in virus growth medium at 37°C, 5% CO2 for 4

days. The cells were then fixed with 10% formalin and stained with 0.1% crystal violet to visualize virus induced cell cytopathic effect (CPE). The viral titer was calculated as $TCID_{50}$/g lung tissue using the Reed-Muench method [33] and estimated the corresponding PFU using the conversion PFU = 0.7 $TCID_{50}$ [73].

Histology and microscopic examination of lung pathology

The left lung was dissected out and fixed in 10% neutral-buffered formalin at room temperature for 24 h, processed in the Leica ASP300S tissue processor, embedded in paraffin, 6 μm thick sagittal sections were cut and stained with H&E for analysis with a bright field microscope. A semi-quantitative scoring system developed previously in our laboratory as described elsewhere [29,30] was used for assessing lung pathology. Blinded visual scoring of individual slides were done on a scale from 0 to 4 for each of the six criteria: parenchymal edema, perivascular edema, degree of bronchial epithelial necrosis, parenchymal inflammatory infiltrates, perivascular inflammatory infiltrates, and alveolar septal wall damage.

RNA extraction and cDNA generation

Lung tissue from individual mice was homogenized in 1 mL TRIzol reagent (ThermoFisher Scientific, cat. no. 15596026) using TissueLyser II (Qiagen, cat. no. 853000) as described elsewhere [29,30]. Genomic DNA traces were removed by treatment with RQ1 RNase-free DNase (Promega, cat. no. M6101) and the RNA concentration was determined using a Nanodrop ND-1000 spectrophotometer (ThermoFisher Scientific). cDNA was synthesized from 2 μg of RNA in a 20 μL volume using RevertAid first strand cDNA synthesis kit (ThermoFisher Scientific, cat. no. K1622) and incubated for 5 min at 25°C followed by 60 min at 42°C. cDNA samples were diluted with 20 μL nuclease-free water and were stored at -20°C until further use.

Quantitative reverse transcription real-time polymerase chain reaction (qRT-PCR)

qRT-PCR was performed using a 10 μL PCR reaction mixture prepared by mixing 5 μL 2× PowerUp SYBR green master mix (ThermoFisher Scientific, cat. no. A25742), 0.5 μM gene- specific forward and reverse primers (listed below in the table), 1 μL of cDNA template, and 3 μL nuclease-free water in the QuantStudio 3 (ThermoFisher Scientific). The following conditions were used: uracil-DNA glycosylase (UDG) activation at 50°C for 2 min, initial denaturation at 95°C for 2 min, and 40 cycles of denaturation and annealing/extension at 95°C for 3 sec and 60°C for 30 sec, respectively. The relative gene expression was calculated with the delta-delta Ct method [74] using ubiquitin C (UBC) for normalization. Results are reported as the fold-change relative to gene expression in mock-treated mice using the delta-delta Ct method [74].

List of primers used in this study are as follows:

Gene		Primer sequence (5'–3')
UBC	Forward	AGGTCAAACAGGAAGACAGACGTA
	Reverse	TCACACCCAAGAACAAGCACA
CCL2	Forward	TTAAAAACCTGGATCGGAACCAA
	Reverse	GCATTAGCTTCAGATTTACGGGT
CCL5	Forward	GCTGCTTTGCCTACCTCTCC
	Reverse	TCGAGTGACAAACACGACTGC
CXCL10	Forward	TGAGTGGGACTCAAGGGATCC
	Reverse	TTCAAGCTTCCCTATGGCCC
TNF	Forward	ACTTCGGGGTGATCGGTCCCC
	Reverse	CCACTTGGTGGTTTGCTACGACGT
IL-6	Forward	TAGTCCTTCCTACCCCAATTTCC
	Reverse	TTGGTCCTTAGCCACTCCTTC
IL-1β	Forward	GCAACTGTTCCTGAACTCAACT
	Reverse	ATCTTTTGG GGT CCG TCA ACT
IL-12p40	Forward	AGACCCTGCCCATTGAACTG
	Reverse	CGGGTCTGGTTTGATGATGTC

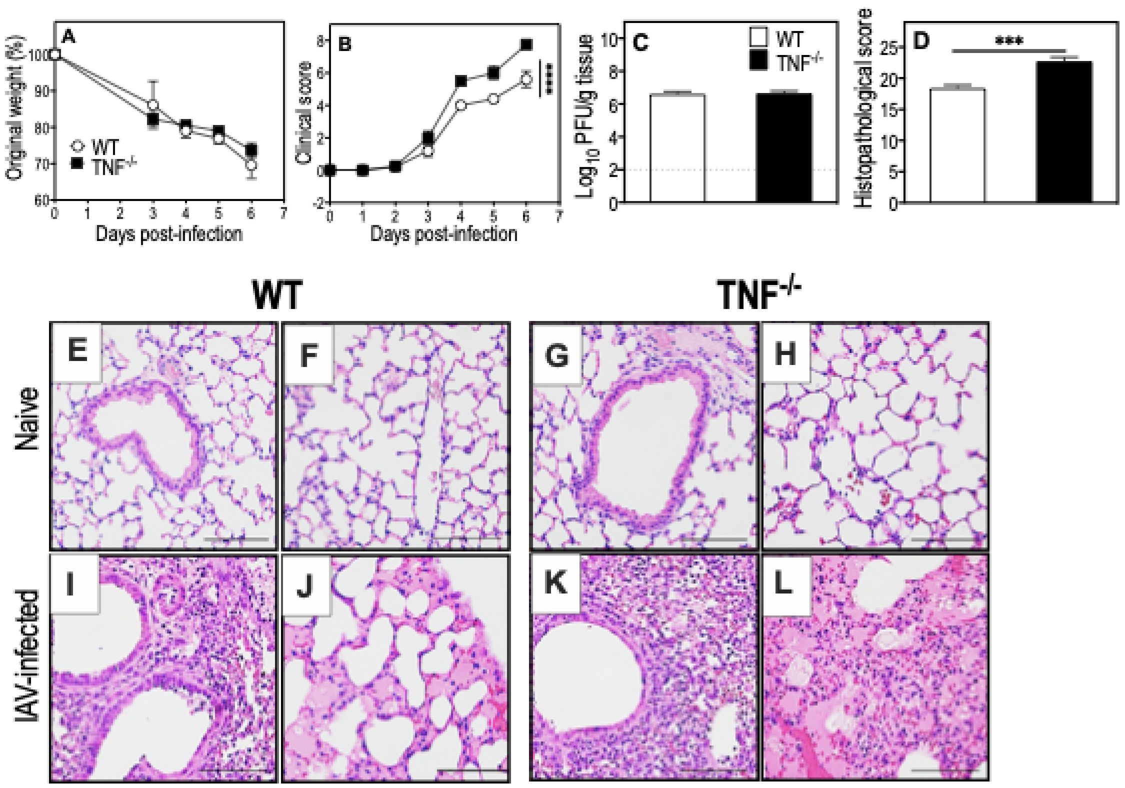

Figure S1. TNF deficiency exacerbates clinical signs and lung pathology in IAV-infected mice. Age-matched groups of female WT and TNF-/- mice (n = 4 or 5) were infected with 3000 PFU IAV i.n. Weight loss and clinical scores (A and B) were assessed until day 6 p.i. when animals were killed. Lungs were collected for various analyses. Lung viral load (C) data were log-transformed and histopathological scores (D) were based on microscopic examination of lung histology H&E sections (E-P), which show that edema, leukocyte infiltration and alveolar septa damage are higher in the lungs of IAV-infected TNF-/- deficient mice compared to WT mice. H&E sections were examined using bright field microscope on all fields at 400× magnification. Data are expressed as means ± SEM and were analyzed using two-way ANOVA with Sidak's post-tests for (A) and (B) and unpaired t-test for (C) and (D). ***, $p \leq 0.001$ and ****, $p \leq 0.0001$. Broken line in panel C corresponds to the limit of virus detection. Bars in panels E-P correspond to 100 μm. Data shown are from a single experiment.

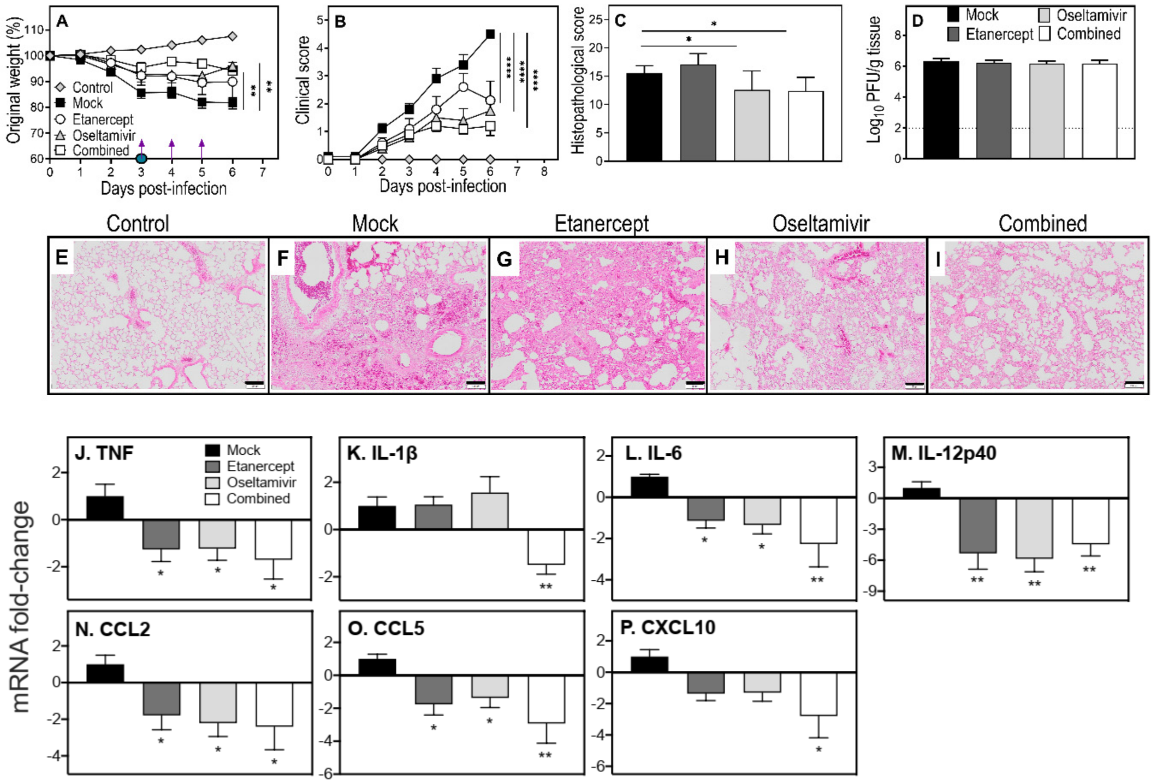

Figure S2. One dose of etanercept combined with standard dose oseltamivir reduces weight loss, clinical scores, lung pathology and mRNA transcripts for pro-inflammatory cytokines/chemokines but not viral load in IAV-infected WT mice. Age-matched groups of female WT mice (n = 5) were infected with 3000 PFU of IAV i.n. Animals were treated with etanercept, oseltamivir (20mg/kg, twice a day), or both drugs (combined) on day 3 p.i. Oseltamivir treatment was continued on days 4 and 5 p.i. as indicated in panel A, where a purple arrow and a filled blue circle symbols indicate oseltamivir and etanercept treatment day, respectively. Animals were killed on day 6 p.i. and lung tissue collected for various analyses. Weight loss (A) and clinical scores (B) were monitored until day 6 p.i. Histopathological scores (C) were derived from microscopic examination of the lung histology H&E sections (E-I), where sections were examined using bright field microscope on all fields at 200× magnification. Viral load (D) data was log-transformed. Lung tissue collected for quantifying mRNA expression levels for specific cytokines and chemokines using qRT-PCR (J-P). Data are expressed as means ± SEM and were analyzed using two-way ANOVA (A and B) or one-way ANOVA (C and D; J-P) with Holm-Sidak's multiple comparisons tests. *, $p < 0.05$; **, $p < 0.01$; and ****, $p < 0.0001$. Broken line in panel D corresponds to the limit of virus detection. Bars in panels E-I correspond to 100 µm.
